# Non-Gonorrhoeal Urethritis

**Published:** 1915-05

**Authors:** 


**Non-Gonorrhoeal Urethritis.** Chajes, Dermat. Zentralblatt. April, 1914. 12 cases were encountered in a series of 96 of urethritis. In one group, the incubation is slow, the course chronic and while presenting scanty subjective and objective symptoms, obstinate. In another, the incubation is short and the symptoms acute but both groups are sterile. In the third group, inflammative bacteria (not gonococci) are found. Sometimes it is possible to isolate the same germ in the male and in the female donor.

